# A Benchmarking Between Deep Learning, Support Vector Machine and Bayesian Threshold Best Linear Unbiased Prediction for Predicting Ordinal Traits in Plant Breeding

**DOI:** 10.1534/g3.118.200998

**Published:** 2019-01-02

**Authors:** Osval A. Montesinos-López, Javier Martín-Vallejo, José Crossa, Daniel Gianola, Carlos M. Hernández-Suárez, Abelardo Montesinos-López, Philomin Juliana, Ravi Singh

**Affiliations:** * Facultad de Telemática; ** Facultad de Ciencias, Universidad de Colima, Colima, Colima, 28040, México; † Departamento de Estadística, Universidad de Salamanca, c/Espejo 2, Salamanca, 37007, España; ‡ International Maize and Wheat Improvement Center (CIMMYT), Apdo. Postal 6-641, 06600, Ciudad de México, México; § Departments of Animal Sciences, Dairy Science, and Biostatistics and Medical Informatics, University of Wisconsin-Madison, Madison, Wisconsin 53706; †† Departamento de Matemáticas, Centro Universitario de Ciencias Exactas e Ingenierías (CUCEI), Universidad de Guadalajara, 44430, Guadalajara, Jalisco, México

**Keywords:** threshold, GBLUP, deep learning, support vector machine, genomic selection, plant breeding, Genomic Prediction, GenPred, Shared Data Resources

## Abstract

Genomic selection is revolutionizing plant breeding. However, still lacking are better statistical models for ordinal phenotypes to improve the accuracy of the selection of candidate genotypes. For this reason, in this paper we explore the genomic based prediction performance of two popular machine learning methods: the Multi Layer Perceptron (MLP) and support vector machine (SVM) methods *vs.* the Bayesian threshold genomic best linear unbiased prediction (TGBLUP) model. We used the percentage of cases correctly classified (PCCC) as a metric to measure the prediction performance, and seven real data sets to evaluate the prediction accuracy, and found that the best predictions (in four out of the seven data sets) in terms of PCCC occurred under the TGLBUP model, while the worst occurred under the SVM method. Also, in general we found no statistical differences between using 1, 2 and 3 layers under the MLP models, which means that many times the conventional neuronal network model with only one layer is enough. However, although even that the TGBLUP model was better, we found that the predictions of MLP and SVM were very competitive with the advantage that the SVM was the most efficient in terms of the computational time required.

Plant breeding is a key scientific area for increasing the food production required to feed the people of our planet. The key step in plant breeding is selection, and conventional breeding is based on phenotypic selection. Breeders choose good offspring using their experience and the observed phenotypes of crops, so as to achieve genetic improvement of target traits (Wang *et al.* 2018). Thanks to this area (and related areas of science), the genetic gain nowadays has reached a near-linear increase of 1% in grain yield yearly (Oury *et al.* 2012; Fischer *et al.* 2014). However, a linear increase of at least 2% is required to cope with the 2% yearly increase in the world population, which relies heavily on wheat products as a source of food (FAO, 2011). For this reason, genomic selection (GS) is now being implemented in many plant breeding programs around the world. GS consists of genotyping (markers) and phenotyping individuals in the reference (training) population and, with the help of statistical models, predicting the phenotypes or breeding values of the candidates for selection in the testing (evaluation) population that only were genotyped. GS is revolutionizing plant breeding because it is not limited to traits determined by a few major genes and allows using a statistical model to establish the associations between markers and phenotypes and also to make predictions of unphenotyped individuals that help do a more comprehensive and reliable selection of candidate individuals. In this way, it is essential for accelerating genetic progress in crop breeding.

Evidence that GS has the potential to revolutionize plant breeding continues to grow. For example, Bernardo and Yu (2007) and Heffner *et al.* (2010) found evidence of the higher genetic gain of GS compared to marker-assisted selection. Also, Albrecht *et al.* (2011) found that GS is superior in terms of genetic gain compared to conventional pedigree breeding. Kadam *et al.* (2016) and Beukert *et al.* (2017) showed that GS is even more efficient that conventional breeding when applied to hybrid crop breeding, because hybrid genotypes can be inferred from their inbred parents, leading to lower genotyping costs (Wang *et al.* 2018). Even in prolific species where not so much gain is expected when implementing GS, Cleveland and Hickey (2013) and Lillehammer *et al.* (2013) showed that GS can be cost-effective in pig breeding and estimated an increase in genetic progress of about 10% when using GS to breed pigs in Norway’s national program. For these reasons, GS has met with a lot of enthusiasm and, since GS has the potential to predict the breeding values of selection candidates at birth more accurately than the classic pedigree index, some breeding companies are re-designing their breeding programs. Consequently, animals (or plants) can be selected at an early age; in some cases, this is expected to double the rate of genetic improvement per year.

Currently, GS is a potent, attractive and valuable plant breeding approach. This method will be integrated into many practical breeding programs in the near future with further advances and the maturing of its theory (Nakaya and Isobe 2012). However, one of the key elements for increasing the power and efficiency of GS are the statistical models that are used for predicting the phenotypes or breeding values of individual candidates for selection. For this reason, there is currently an extensive area of research aimed at improving existing models and developing new models in order to increase the precision of candidate selection using GS.

However, this task is challenging since specific models are needed for each type of response variable (phenotype). In two recent pioneer articles, Montesinos-López *et al.* (2018a, b) evaluated the prediction performance of univariate and multivariate neural network deep learning models for continuous response variables. In general, much more research had been done for quantitative (continuous) traits than for ordinal, binary and count traits. Categorical scores for disease susceptibility or resistance often are recorded in plant breeding. For example ordinal traits are very common in plant breeding programs for measuring disease incidence and severity, for sensory evaluation, such as perceived quality of a product (*e.g.*, taste, smell, color, decay), and plant development (*e.g.*, developmental stages, maturity). These types of data are often partially subjective since the scale indicates only relative order and not absolute amounts; therefore the intervals between successive categories might not be the same (Simko and Piepho 2011).

Montesinos-López *et al.* (2015a) introduced genomic models for analyzing ordinal characters and to assess the genomic based for ordered categorical phenotypes using a threshold model that is the counterpart of the Genomic Best Linear Unbiased Predictor (*i.e.*, TGBLUP). The threshold model TGBLUP relates hypothetical underlying scale to the outward categorical response and it was extended to account for genomic × environment interactions. The models that included G×E achieved up to 14% more gains in prediction accuracy as compared with the main effect models. Montesinos-López *et al.* (2015b) implemented Bayesian logistic ordinal regression in the context of genomic-enabled prediction using the Pólya-Gamma data augmentation approach that produces a Gibbs sampler with similar full conditional distributions of the Bayesian probit ordinal regression.

Machine Learning (ML) methods have been proposed in the academic literature as alternatives to statistical methods for predicting phenotypes or breeding values in the context of GS. ML has gained considerable prominence over the last decade fueled by a number of high profile applications in Autonomous Vehicles, intelligent robots, image and speech recognition, automatic translations, medical and law usage, as well as for beating champions in games like chess, Jeopardy, GO and poker (Makridakis *et al.* 2018). ML also has high profile applications in biological science research (genomics, proteomics or metabolomics) to extract features, functions, structure and molecular dynamics from raw biological sequence data (*e.g.*, DNA, RNA, and amino acids). Alipanahi *et al.* (2015) used deep learning to predict DNA- and RNA-binding proteins. Zhang *et al.* (2016) developed a deep neural network framework to model structural features of restricted Boltzmann machines. Pan and Shen (2017) proposed a hybrid convolutional neural network-deep belief network model to predict restricted Boltzmann machine interaction sites and motifs on RNAs. Quang *et al.* (2015) proposed a deep neural network model to annotate and identify pathogenicity in genetic variants.

For stem rust in wheat, Ornella *et al.* (2012) analyzed and compared the performance of Bayesian Lasso, ridge regression, and support vector machine. Bayesian Lasso and ridge regression had slightly superior prediction accuracy than support vector regression.

Ornella *et al.* (2014) evaluated six regression models, Bayesian Lasso, ridge regression, random forest regression, Reproducing Kernel Hilbert space, and two support vector regression in several wheat rust data bases; the authors found that random forest regression and Reproducing Kernel Hilbert Space were the best models. Recently, in a very comprehensive review, González-Camacho *et al.* (2018), presented and discussed several ML methods applied in genomic selection to predict rust resistance in wheat as well as classifications and regression methods. These authors compared results from linear models with those from ML, random forest, support vector machine (SVM) and radial basis function neural network and they found that in general the SVM with linear kernel was the best in terms of genomic based prediction performance (González-Camacho *et al.* 2018).

As stated above, ML methods are applied in many domains of science and technology. However, it is still not clear if ML methods outperform conventional statistical models in terms of prediction performance, since there is only weak empirical evidence of the relative performance of ML methods. Most of the time, ML methods are supported by few real data sets, which raises questions about the statistical significance of the results and their generalization (Makridakis *et al.* 2018). No benchmarks are used to compare the accuracy of ML methods *vs.* alternative ones (Makridakis *et al.* 2018). For this reason, it is of paramount importance to objectively evaluate the relative performance of ML methods in terms of prediction performance as compared to the conventional GS models in order to improve prediction accuracy and the selection of candidate genotypes early in time. For these reasons, in this paper we compare the conventional GS model TGBLUP with two popular models of the machine learning domain –MLP and SVM models − with the goal of exploring its prediction accuracy and practical implementation in the GS context for ordinal traits. We compare the prediction performance of the three models with seven real data sets and cross-validation, using the percentage of cases correctly classified (PCCC) as a metric.

## Material and Methods

### Implemented models

#### Bayesian threshold genomic best linear unbiased prediction (TGBLUP):

We used $y_{ij}$ to represent the ordinal response, that belong to exactly one of the C mutually exclusive categories, of the jth line in the ith environment with i=1,...,I;j=1,2,...,J and we propose the following C−1 linear predictors to fully specified the model related to the C response probabilities that takes into account the genotype × environment (G×E) interaction term:

$$\eta_{ij(1)}={\text{Φ}}^{-1}\left(\pi_{ij\left(1\right)}\right)=\gamma_{1}-E_{i}-g_{j}-gE_{ij}$$ (1)

$$\eta_{ij(2)}={\text{Φ}}^{-1}\left(\pi_{ij\left(1\right)}+\pi_{ij\left(2\right)}\right)=\gamma_{2}-E_{i}-g_{j}-gE_{ij}$$ (2)

...

$$\eta_{ij(C-1)}={\text{Φ}}^{-1}\left(\pi_{ij\left(1\right)}+\text{...}+\pi_{ij\left(C-1\right)}\right)=\gamma_{C-1}-E_{i}-g_{j}-gE_{ij}$$ (3)

where $\eta_{ij(c)}$ denotes the c^th^ predictor (c=1,2,...,C−1) for the fixed and random effects combination, $\pi_{ij(c)}$ represents the probability in environment i, line j in category c; and $\gamma_{c}$ is the threshold (intercept) for the c^th^ predictor. $E_{i}$ represents environment *i* and is assumed fixed, $g_{j}$ is the marker effect of genotype *j*, and $gE_{ij}$ is the G×E interaction term. Distributions: $y_{ij(1)},y_{ij(2)},\text{...},y_{ij(C)}|E_{i},g_{j},gE_{ij}∼$Multinomial $(\pi_{ij(1)},\pi_{ij(2)},\text{...},\pi_{ij(C)})$. Only C−1 link functions and cummulative probabilities are needed (estimated) since the cumulative probability of the last category C is 1 and having the first C−1 probabilities we can obtain the probability of the last category. $b_{1}={\left(g_{1},\text{...},g_{J}\right)}^{T}∼N\left(0,G_{1}\sigma_{g}^{2}\right)$, where $G_{1}$ is the Genomic Relationship Matrix (GRM) that is calculated as $G_{1}=\frac{WW^{T}}{m}$ (as proposed by VanRaden 2008), where W is a matrix of scaled markers alleles of dimension J×m, $\sigma_{g}^{2}$ is the genotypic variance and m denotes the number of markers. The $G_{1}\;$ matrix is a covariance matrix that contains the similarity between individuals based on marker information, rather than the expected similarity based on pedigree. $b_{2}={\left(gE_{11},\text{...},gE_{IJ}\right)}^{T}∼N\left(0,G_{2}\sigma_{gE}^{2}\right)$, where $G_{2}$ is computed as $G_{2}=I_{I}⊗G_{1}$ of order *IJ*x*IJ* and ⊗ denotes the Kronecker product, $I_{I}\;$ means that we assume independence between environments (Montesinos-López *et al.* 2015a,b) and $\sigma_{gE}^{2}$ is the variance corresponding to the G×E interaction term. $G_{1}$ and $G_{2}$ were assumed known. Link function: cumulative probit: Φ(.) is the cumulative distribution function of a standard normal distribution (probit link) and ${\text{Φ}}^{-1}$ its corresponsing inverse. For this model, the inverse link is as follows: $\pi_{ij\left(1\right)}=\text{Φ}\left(\eta_{ij\left(1\right)}\right),\;\pi_{ij\left(1\right)}+\pi_{ij\left(2\right)}=\text{Φ}\left(\eta_{ij\left(2\right)}\right),\text{...},\pi_{ij\left(1\right)}+\pi_{ij\left(2\right)}+\text{...}+\pi_{ij\left(C-1\right)}=\text{Φ}\left(\eta_{ij\left(C-1\right)}\right)$. Once we have estimates of $\text{Φ}\left(\eta_{ij\left(1\right)}\right),$ $\text{Φ}\left(\eta_{ij\left(2\right)}\right),\text{...},\text{ Φ}\left(\eta_{ij\left(C-1\right)}\right),$ we can estimate $\pi_{ij\left(2\right)}=\text{Φ}\left(\eta_{ij\left(2\right)}\right)-\text{Φ}\left(\eta_{ij\left(1\right)}\right)$, $\pi_{ij\left(3\right)}=\text{Φ}\left(\eta_{ij\left(3\right)}\right)-\text{Φ}\left(\eta_{ij\left(2\right)}\right),\text{...},$ $\pi_{ij\left(C\right)}=1-\text{Φ}\left(\eta_{ij\left(C-1\right)}\right)$ (Montesinos-López *et al.*, 2015a). This threshold model assumes that the process that gives rise to the observed categories is an underlying continuous variable with a normal distribution $l_{ij}=E_{i}+g_{j}+gE_{ij}+\epsilon_{ij}$; where $l_{ij}$ are called “liabilities,” $\epsilon_{ij}∼N(0,1)$ (*e.g.*, Gianola, 1982, and Sorensen *et al.* 1995) and the ordinal categorical phenotypes with C categories are generated from the underlying phenotypic values, $l_{ij}$, as $y_{ij}=1$ if $-\infty <l_{ij}<\gamma_{1};\;y_{ij}=2$ if $\gamma_{1}<l_{ij}<\gamma_{2};$ ....$,\;y_{ij}=C$ if $\gamma_{C-1}<l_{ij}<\infty$. The implementation of the TGBLUP model was done in the BGLR package of de los Campos and Pérez-Rodríguez (2014) in the R statistical software (R Core Team 2018).

#### Multi Layer Perceptron (MLP) for ordinal data:

**Figure 1:**
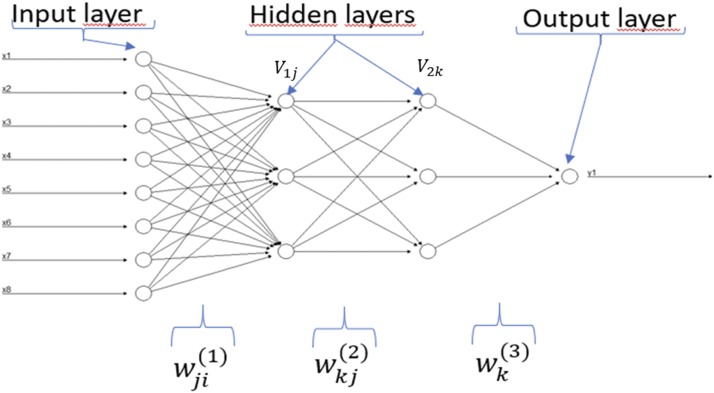
Example of a feedforward neural network with eight input variables (x1,..,x8), one output variable (y1) and two hidden layers with three neurons each. The input variables correspond to information of environments, genotypes and genotype×environment interaction. The marker information is included in the genotype and genotype×environment interaction through the Cholesky decomposition of the genomic relationship matrix. The output variable is the ordinal response variable that we are interested to predict.

The architecture of the deep learning method we implemented is depicted in Figure 1; this architecture is called densely connected network or feedforward neural network, since it consists of an input layer, an output layer (for univariate-trait modeling) and multiple hidden layers between the input and output layers. There are many other deep learning architectures (convolutional networks, recurrent networks, etc.), which can be found in Gulli and Sujit (2017), Angermueller *et al.* (2016) and Chollet and Allaire (2017).

The architecture shown in Figure 1 was applied to seven ordinal real data sets with 1, 2 and 3 hidden layers and number of neurons (from 10 to 500 with increases of 20). The input variables of the MLP model ($x=\{x_{ip}\},$ i = 1,2,..,n; *p* = 1,2,..,$N_{1}$) given in Figure 1 are the result of concatenating the information on environments, the information on markers through the Cholesky decomposition of the genomic relationship matrix and the information on the genotype × environment interaction (G×E). Via weights, the input variables ($x_{ip}$) and the units (neurons) of the hidden layers are connected. The information to the neurons in the first hidden layer is simply passed by the input variables. In the first hidden layer, the net input into the jth hidden unit is $h_{1j}=\overset{N_{1}}{\underset{p=1}{\sum }}w_{jp}^{\left(1\right)}x_{p}+b_{j}^{\left(1\right)}$, where $N_{1}$ is the total number of input variables, $w_{jp}^{\left(1\right)}$ is the weight of input unit p to hidden unit j in the first hidden layer, $x_{p}$ is the value of the pth input variable and $b_{j}^{\left(1\right)}$ is a bias specific to each neuron in layer 1. Then an activation function is applied to the net input of the jth hidden unit in the first hidden layer and outputs $V_{1j}=g_{1}(h_{1j})$ for $j=1,\text{...},\;N_{2}$. Similarly, the net input that neuron k in the second hidden layer receives is $h_{2k}=\overset{N_{2}}{\underset{j=1}{\sum }}w_{kj}^{\left(2\right)}V_{1j}+b_{k}^{\left(2\right)}$, where $N_{2}$ is the total number of input neurons that come from hidden layer 1 to neuron k, $w_{kj}^{\left(2\right)}$ is the weight from unit j of layer 1 that goes to unit k in hidden layer 2, $V_{1j}$ is the value of the output of unit j in layer 1 and $b_{k}^{\left(2\right)}$ is a bias specific term to neuron k in layer 2. Then an activation function is applied to the net input of the kth hidden unit in the second hidden layer and outputs $V_{2k}=g_{2}(h_{2k})$ for k=1,...,M. Similarly, the unique output unit receives a net input of $h_{3}=\overset{M}{\underset{k=1}{\sum }}w_{k}^{\left(3\right)}V_{2k}+b^{\left(3\right)}$, where M is the number of hidden units from hidden layer 2, and $w_{k}^{\left(3\right)}$ is the weight from hidden unit k in layer 2 to the unique output. Finally, the prediction of individuals for the unique trait is obtained as: $\hat{y}=g_{3}(h_{3})$. It is important to point out that we used the sigmoid and softmax activation functions in the output layer ($g_{3})$ when the response variables were binary and ordinal respectively, since we are working with binary or ordinal phenotypes. However, for the hidden activations functions we implemented the rectified linear activation unit (RELU) function.

The successful implementation of the MLP model depends on appropriately selecting the following hyperparameters: (1) number of units (U), (2) number of layers, (3) number of epochs (E), (4) type of regularization method and (5) type of activation function. An epoch means one pass (forward and backward) of the full training set through the neural network. Regarding the number of units, we used between 10 to 500 units with increases of 20, and with regard to the number of layers we used 1, 2 and 3; we used from 1 to 100 epochs, and the type of regularization we chose was dropout regularization for training the models (Gulli and Sujit 2017; Chollet and Allaire 2017; Srivastava *et al.* 2014). For more details on model selection in MLP models, we suggest reading the papers by Montesinos-López *et al.* (2018a, b), where the authors evaluate the prediction performance of univariate and multivariate deep learning models for continuous response variables. All MLP models were implemented in the keras package (Chollet and Allaire 2017) in the open-source software R (R Core Team 2018).

#### Support vector machine:

Support Vector Machine is one of the most popular and efficient machine learning algorithms, which was proposed to the computer science community in the 1990s by Vapnik (1995) and used mostly for classification problems. Its versatility and the fact that it performs well in the presence of a large number of predictors, even with a small number of cases, makes SVM very appealing for tackling a wide range of problems such as speech recognition, text categorization, image recognition, face detection, faulty card detection, junk mail classification, credit rating analysis, and cancer and diabetes classification, among others (Attewell *et al.* 2015; Byun and Lee 2002). Briefly, SVM is the solution to the optimization problem:

$$\underset{\beta_{0},\;\beta_{11},\;\beta_{12},\ldots ,\;\beta_{p1},\;\beta_{p2},\;\epsilon_{1},\ldots ,\;\epsilon_{n}}{\underset{︸}{maximize}}\;M$$ (4)

$$\text{subject to }\overset{p}{\underset{j=1}{\sum }}\overset{2}{\underset{k=1}{\sum }}\beta_{jk}^{2}=1,$$ (5)

$$y_{i}\left(\beta_{0}+\overset{p}{\underset{j=1}{\sum }}\;\beta_{j1}x_{ij}+\overset{p}{\underset{j=1}{\sum }}\;\beta_{j2}x_{ij}^{2}\right)\geq M\left(1-\epsilon_{i}\right),$$ (6)

$$\epsilon_{i}\geq 0,\;\overset{n}{\underset{i=1}{\sum }}\epsilon_{i}\leq T,$$ (7)

where $\beta_{0},\;\beta_{11},\;\beta_{12},\text{...},\;\beta_{p1},\;\beta_{p2}$ are the coefficients of the maximum margin hyperplane. A hyperplane is a subspace whose dimension is one less than that of its original space. For a space in 3-dimensions its hyperplanes has 2-dimensions, while for a space in 2-dimensions, its hyperplanes is a line (one dimension). T is a non-negative tuning parameter that determines the number and severity of the violations to the margin (and to the hyperplane) that we will tolerate and is seen as the total amount of errors allowed since it is the bound of the sum of $\epsilon_{i}^{\prime }s$. For T close to zero, the soft-margin SVM allows very little error and is similar to the hard-margin classifier (James *et al.* 2013). The larger T is, the more error is allowed, which in turn allows for wider margins. In practice, T is treated as a tuning parameter that is generally chosen via cross-validation. M is the width of the margin and we seek to make this quantity as large as possible. In (7), $\epsilon_{1},\text{...},\;\epsilon_{n}$ are slack (error) variables that allow individual observations to be on the wrong side of the margin or the hyperplane. The slack variable $\epsilon_{i}$ tells us where the ith observation is located, relative to the hyperplane and relative to the margin. If $\epsilon_{i}$=0, then the ith observation is on the correct side of the margin, If $\epsilon_{i}$>0, then the ith observation is on the wrong side of the margin, and we say that the ith observation has violated the margin. If $\epsilon_{i}$>1, then it is on the wrong side of the hyperplane. Once we have solved (4)–(7), we classify a test observation $x^{*}$ by simply determining on which side of the hyperplane it lies. That is, we classify the test observation in the training/testing sets based on the sign of $f(x^{*})$ =$\;\beta_{0}+\beta_{1}x_{1}^{*}+\beta_{2}x_{2}^{*}+\text{...}+\beta_{p}x_{p}^{*}$; if $f(x^{*})$<0, then the observation is assigned to the class corresponding to -1, but if $f(x^{*})$>0, then the observation is assigned to the class corresponding to 1 (James *et al.* 2013). We chose f(x) as a nonlinear function of x and implemented the radial kernel. This type of kernel is a nonlinear function of x, but with fewer parameters than quadratic, cubic, or higher order expansion of x. The SVM with radial kernel [$K\left(x_{i},x_{i\prime }\right)=\text{exp}\left(-\gamma \overset{p}{\underset{j=1}{\sum }}\left(x_{ij}-x_{i\prime j}\right)\right)\text{ with }\gamma \text{ a positive constant }(\text{James}et\;al.,\;2013)]$ was implemented with the R package e1071 in the R statistical software (R Core Team 2018). Also, due to the fact that we work with binary (K = 2 classes) and ordinal (K > 2 classes) data for the ordinal response variables we implemented the one-*vs.*-one classification approach that construct K(K−1)/2 binary SVMs each of which compare a pair of classes. Each SVM compare the k*th* class coded as +1, to the k′*th* class, coded as -1. At prediction time, a voting scheme is applied: all K(K−1)/2 binary SVMs are applied to an unseen sample and the class that got the highest number of “+1” predictions gets predicted by the combined classifier (James *et al.* 2013).

### Experimental data sets

#### Phenotypic data sets:

In this study, we used the data set of Juliana *et al.* (2018). The data used belong to four elite yield trial (EYT) nurseries from the Global Wheat Program of the International Maize and Wheat Improvement Center (CIMMYT). The EYT nurseries were planted in mid-November. They were planted in bed and flat planting systems in optimally irrigated environments and received 500 mm of water at the Norman E. Borlaug Research Station, Ciudad Obregon, Sonora, Mexico. The nurseries were sown in 39 trials, each comprising 28 lines and two high-yielding checks (Kachu and Borlaug) that were arranged in an alpha lattice design with three replications and six blocks. The nurseries were evaluated for the following traits: number of days from germination to 50% spike emergence (days to heading, DTHD), number of days from germination to 50% physiological maturity (days to maturity, DTMT), grain yield (GY, tons per hectare) and plant height (Height, centimeters). All these nurseries were evaluated during four seasons: 2013-2014 (EYT 13-14; here called **data set 1**), 2014-2015 (EYT 14-15; called **data set 2**), 2015-2016 (EYT 15-16; called **data set 3)** and 2016-2017 (EYT 16-17; called **data set 4**). It is important to point out that the trait GY was ignored for this application.

Data set 1 included 767 lines, data set 2,775 lines, data set 3,964 lines, and data set 4, 980 lines (Juliana *et al.* 2018). In addition, in each season we studied six environments resulting from the level of irrigation (IR) and planting system (bed or flat) which we called: Bed2IR, Bed5IR, Flat5IR, FlatDrip, EHT and LHT. However, all these environments were not evaluated in all seasons (data sets). Table 1 gives the environments under study in each of the first four data sets.

**Table 1:** Environments evaluated in data sets 1, 2, 3 and 4. Environments in data set 1 are Bed5IR (bed planting and 5 Irrigation levels), EHT (early heat stress), Flat5I (flat plating system and 5 irrigation levels), LTH (late heat stress). Environments in data set 2 are Bed2IR (bed planting and 2 Irrigation levels), Bed5IR (bed planting and 5 Irrigation levels), EHT (early heat stress), Flat5IR (flat plating system and 5 irrigation level), LTH (late heat stress). Environments in data set 3 are Bed2IR (bed planting and 2 Irrigation levels), Bed5IR (bed planting and 5 Irrigation levels), Flat5IR (flat plating system and 5 irrigation level), FlatDrip (flat planting system a drip irrigation). Environments in data set 4 are Bed5IR (bed planting and 5 Irrigation levels), EHT (early heat stress), Flat5IR (flat plating system and 5 irrigation level), FlatDrip (flat planting system a drip irrigation)

Data set	Environments evaluated
Data set 1	Bed5IR, EHT, Flat5IR and LHT
Data set 2	Bed2IR, Bed5IR, EHT, Flat5IR and LHT
Data set 3	Bed2IR, Bed5IR, Flat5IR and FlatDrip
Data set 4	Bed5IR, EHT, Flat5IR and FlatDrip.

It is important to point out that here we used the BLUEs of each of the lines obtained (as suggested by Juliana *et al.* 2018) adjusted for trials, blocks and replications in each data set. The three traits used were discretized because the original data sets are continuous, only to illustrate the proposed models. Traits DTHD and DTMT were discretized at quantiles 33.33% and 66.67% (in **data sets 1** and **2**) to obtain three categories, while trait Height was discretized at quantile 50% to obtain 2 categories (in **data sets 1**, **2**, **3** and **4**); the discretization process was done for each environment of each data set. For **data sets 3** and **4**, traits DTHD and DTMT were discretized at quantiles 20%, 45%, 70% and 90%.

**Data set 5** is part of **data set 3**; for this reason, the phenotypic information and genomic information were obtained in the same way as in **data set 3**; however, only 964 lines had complete data on the total 980 lines under study in **data set 3.** But now the traits measured in **data set 5** were grain color (GC) (1 = yes, 2 = no), leaf rust (ordinal scale with 5 points), stripe rust (ordinal scale with 3 points) and GY, which is a continuous trait (this trait was not used because it is continuous). **Data set 6** and **data set 7** are part of the wheat yield trial (YT) nurseries from CIMMYT’s Global Wheat Breeding Program. For **data set 6**, the number of lines used was 945 and for **data set 7**, 1145 wheat lines were used. A continuous trait (grain yield, GY) and an ordinal trait (lodging, ordinal scale of 5 points) were measured on both data sets. However, only lodging was used in this paper due to the fact that the other trait is continuous.

#### Genotypic data:

The lines used in this study were 3,486 (79.807%) out of 4,368 lines that were evaluated in the four seasons (nurseries) comprising the **data sets 1**, **2**, **3**, and **4** were genotyped using genotyping-by-sequencing (GBS) (Elshire *et al.* 2011; Poland *et al.* 2012) at Kansas State University, using an Illumina HiSeq2500 for obtaining genome-wide markers. Marker polymorphisms were called across all lines using the TASSEL (Trait Analysis by Association Evolution and Linkage) GBS pipeline (Glaubitz *et al.*, 2014) and anchored to the International Wheat Genome Sequencing Consortium’s (IWGSC) first version of the reference sequence (RefSeq v1.0) assembly of the bread wheat variety Chinese Spring. Markers with more than 60% missing data, less than 5% minor allele frequency and percent heterozygosity greater than 10% were removed; as a result, we obtained 2,038 markers. Missing marker data were imputed using LinkImpute (Money *et al.* 2015) implemented in TASSEL (Bradbury *et al.* 2007), version 5. The lines were also filtered for more than 50% missing data and we end up with 3,486 lines (79.807%) of the total 4,368 lines originally evaluated (767 lines from **data set 1**, 775 lines from **data set 2**, 964 lines from **data set 3** and 980 lines from **data set 4**) (Juliana *et al.* 2018). The lines used in **data sets 5**, **6**, and **7** were genotyped with the same marker system that was used for the other data sets.

### Evaluation of prediction accuracy with cross-validation

The prediction accuracy of the three models under study (TGBLUP, MLP and SVM) was evaluated with an outer cross-validation (CV), while the prediction accuracy of the MLP and SVM models, in addition to outer cross-validation, was also evaluated using an inner cross-validation as was done in Montesinos-López *et al.* (2018a, b). The outer CV was used for evaluating the prediction accuracy of the three models, while the inner CV was used for tuning the hyperparameters in the MLP and SVM models. In the outer CV, the original data set was partitioned into five subsamples of equal size and each time four of them were used for training (TRN) and the remaining one for testing (TST), that is, we implemented a fivefold cross-validation. In the design, some lines can be evaluated in some, but not all, target environments, which mimics a prediction problem faced by breeders in incomplete field trials. Our cross-validation strategy is the same as the strategy denoted as CV2 that was proposed and implemented by Jarquín *et al.* (2017), where a certain portion of test lines in a certain portion of test environments is predicted, since some test lines that were evaluated in some test environments are assumed to be missing in others. We used the percentage of cases correctly classified (PCCC) for evaluating the prediction performance since our response variables were binary and ordinal and it was calculated from each trait-environment combination for each of the testing sets and the average of all folds was reported as a measure of prediction performance. It is important to point out that, to avoid biased results, the tuning step was done in each fold using only the training set. As mentioned above, we implemented an inner CV for the MLP and SVM models.

As mentioned above, for the MLP and SVM methods we implemented an inner CV using the grid search method. For the MLP model, the grid for the number of epochs and units was explained above, where the MLP was presented and each training set of the outer CV was split, with 20% of the data in the inner testing set and the remaining 80% in the inner training set. This inner CV was implemented using the validation_split argument on the fit function of the keras library to avoid implementing manual k-fold cross-validation for the inner CV, which requires more computational resources (Chollet and Allaire 2017). However, for the SVM method, the inner cross-validation was implemented with a 10-fold cross-validation with the following values for parameters T and γ: T = (1,1.2,1.4,1.6,1.8,2) and γ=(0.0001,0.0002,0.00025,0.0003). Then the outer CV was implemented with the best combination of T and γ in each training set.

### Data availability

Details of the phenotypic and genomic data of the seven data sets used in this study can be downloaded in the link: http://hdl.handle.net/11529/10548140. The seven data sets have also trait grain yield (not used in this study). These seven data sets were also used in Montesinos-López *et al.* (2018c, Manuscript submitted for publication) including trait grain yield.

## Results

The results are given in seven sections, one for each data set under study. Each section gives a descriptive analysis of each data set and the prediction accuracy obtained for each of the three models that we implemented. Also Table A1 (Appendix) shows the Average percentage of cases correctly classified for each data set (data sets 1-7), model (SVM, TGBLUP and MLP), layer, type of interaction terms with genotype × environment (I) and without genotype × environment (WI), and trait.

### Data set 1

**Figure 2:**
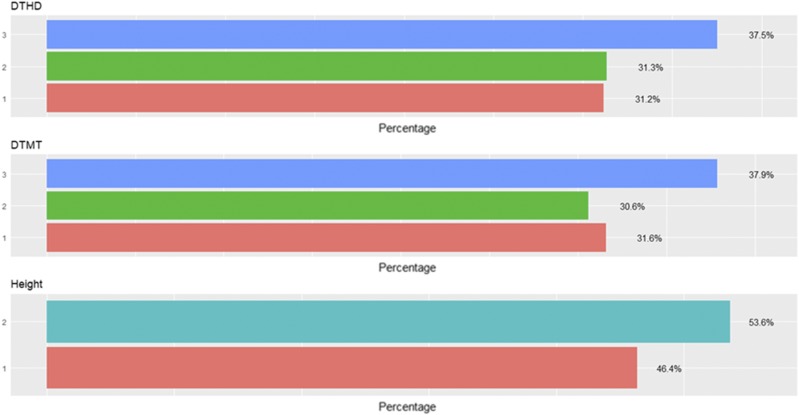
Percentage of individuals in each category of the ordinal response for *data set 1* across environments for traits days to heading (DTHD), days to maturity (DTMT) and Height.

**Figure 3:**
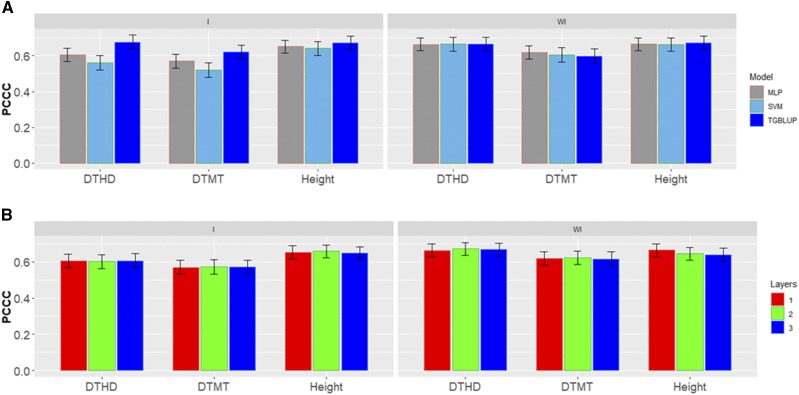
*Data set 1* in terms of percentage of cases correctly classified (PCCC) for traits days to heading (DTHD), days to maturity (DTMT) and Height. (A) Prediction accuracy of TGBLUP, MLP with one layer and SVM models with the G×E term (I) and without the G×E term (WI) for each trait; (B) prediction accuracy with different numbers of layers (1, 2 and 3) across environments with the MLP model with the G×E term (I) and without the G×E term (WI).

Figure 2 shows the percentages of individuals in each category for each trait where can be observed that the number of individuals in each category are different. Figure 3 gives the prediction accuracies for the three traits under the three methods (TGBLUP, MLP and SVM) with (I) and without (WI) the genotype × environment interaction. The best predictions with the interaction term (I) were observed under the TGBLUP model and the worst occurred under the SVM method and the range of predictions with (I) was between 0.5206 and 0.6773. Without the interaction term (WI), we did not find statistical differences between the three methods (TGBLUP, SVM and MLP) for the three traits under study, and the range of predictions was between 0.5992 and 0.6722. Figure 3 also provides the predictions for each trait with (I) and without (WI) the interaction term using 1, 2 and 3 layers under the MLP model. The Figure 3 shows (subpanel b) that there are no statistical differences between the number of layers used (exist overlapping of the corresponding confidence intervals), which was not expected, since with deep learning methods, using more deep hidden layers helps to capture complex interactions which many times help to increase prediction accuracies.

### Data set 2

**Figure 4:**
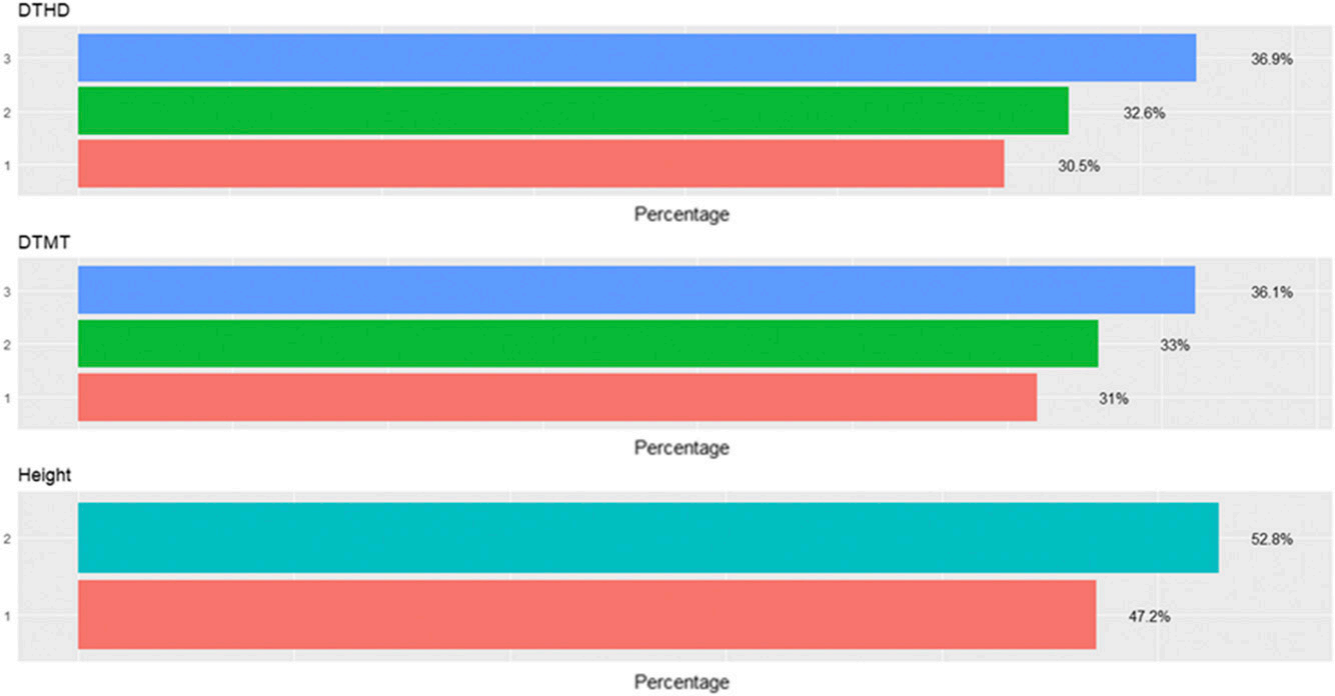
Percentage of individuals in each category of the ordinal response for *data set 2* across environments for traits days to heading (DTHD), days to maturity (DTMT) and Height.

**Figure 5:**
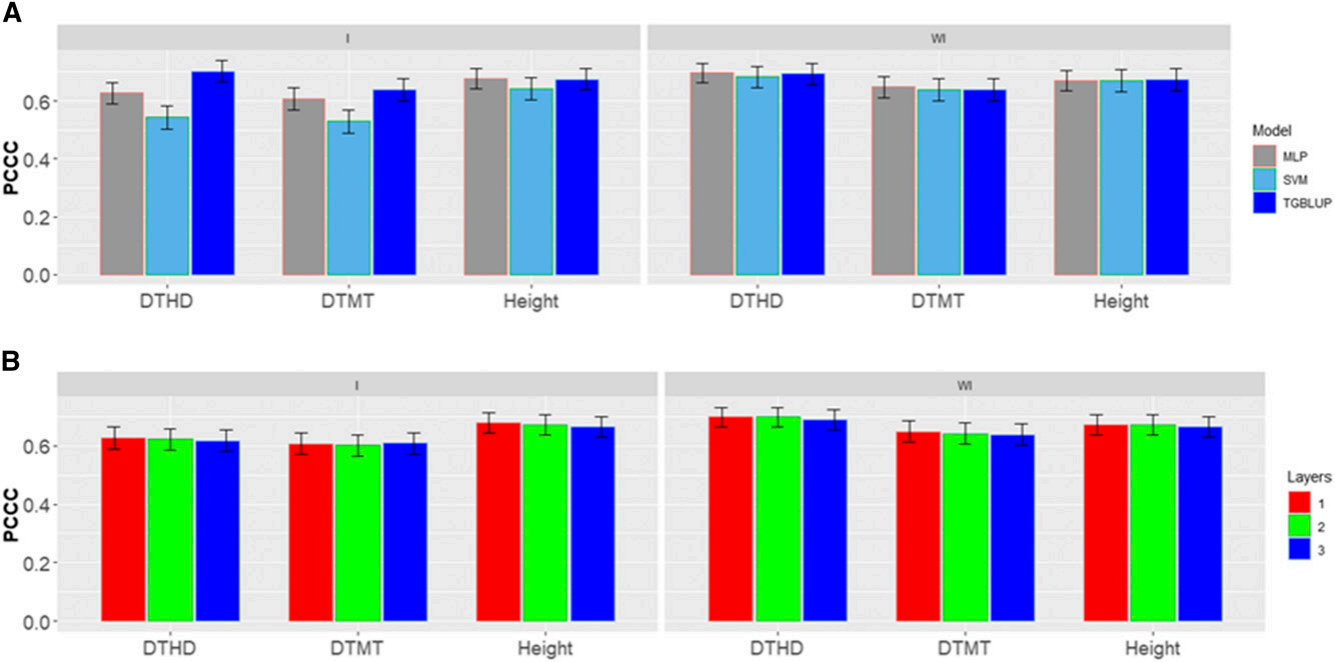
*Data set 2* in terms of percentage of cases correctly classified (PCCC) for traits days to heading (DTHD), days to maturity (DTMT) and Height. (A) Prediction accuracy of TGBLUP, MLP with one layer and SVM models with the G×E term (I) and without the G×E term (WI) for each trait; (B) prediction accuracy with different numbers of layers (1, 2 and 3) across environments with the MLP model with the G×E term (I) and without the G×E term (WI).

The percentages of individuals in each category for data set 2 are given in Figure 4, were we can see that each category has a different number of individuals. Figure 5 gives the predictions in terms of PCCC for the three methods under study for data set 2, which consists of three traits. The predictions are provided with genotype × environment interaction (I) and without genotype × environment interaction (WI). The predictions obtained with (I) ranged between 0.5297 and 0.7021. The best predictions with the interaction (I) term were observed under the TGBLUP model, and the worst under the SVM method. Without the interaction term (WI), the predictions ranged between 0.6371 and 0.6970, and we found no statistical differences between the three methods (TGBLUP, SVM and MLP) (Figure 5). We also did not find statistical differences between the MLP models using 1, 2 and 3 layers, which means that the conventional neural network (with one hidden layer) is as good as the deep learning models that had two or three hidden layers.

### Data set 3

**Figure 6:**
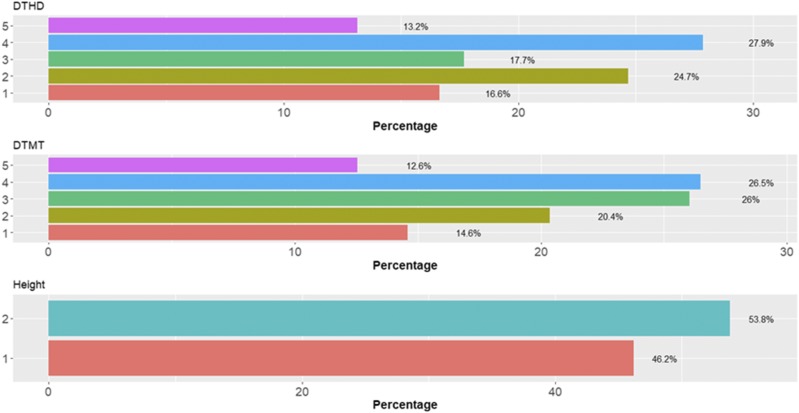
Percentage of individuals in each category of the ordinal response for *data set 3* across environments for traits days to heading (DTHD), days to maturity (DTMT) and Height.

**Figure 7:**
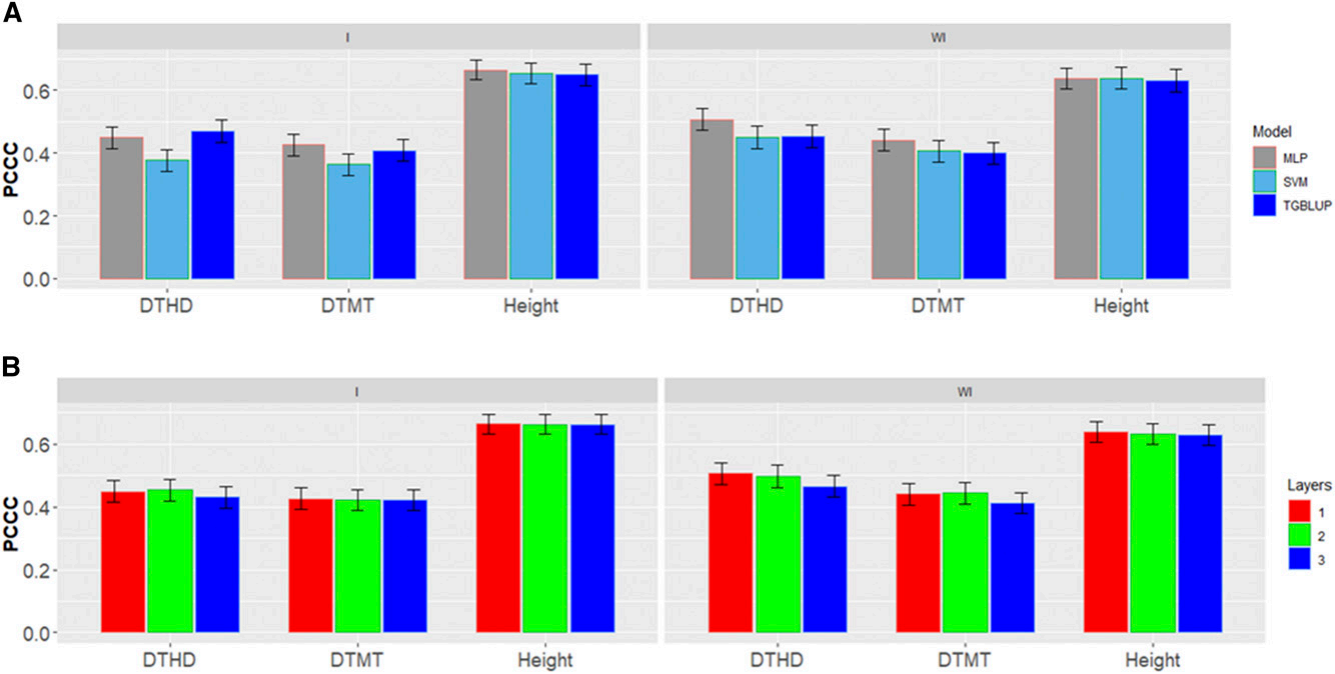
*Data set 3* in terms of percentage of cases correctly classified (PCCC) for traits days to heading (DTHD), days to maturity (DTMT) and Height. (A) Prediction accuracy of TGBLUP, MLP with one layer and SVM models with the G×E term (I) and without the G×E term (WI) for each trait; (B) prediction accuracy with different numbers of layers (1, 2 and 3) across environments with the MLP model with the G×E term (I) and without the G×E term (WI).

We can observe that there are different percentages of individuals in each category in data set 3 (Figure 6). The prediction accuracies in terms of PCCC for the three traits in this data set are given for the three methods evaluated with (I) and without (WI) the genotype× environment interaction term (Figure 7). When the genotype× environment interaction term was taken into account, the predictions ranged between 0.3630 and 0.6636, and the TGBLUP model was the best. On the other hand, when the genotype× environment interaction term was ignored, the predictions ranged between 0.3998 and 0.6377, and the best predictions occurred under the MLP method in two out of the three traits; however, the MLP was not statistically superior to the TGBLUP model. Finally, we found no significant differences using 1, 2 and 3 layers with the deep learning methods (Figure 7).

### Data set 4

**Figure 8:**
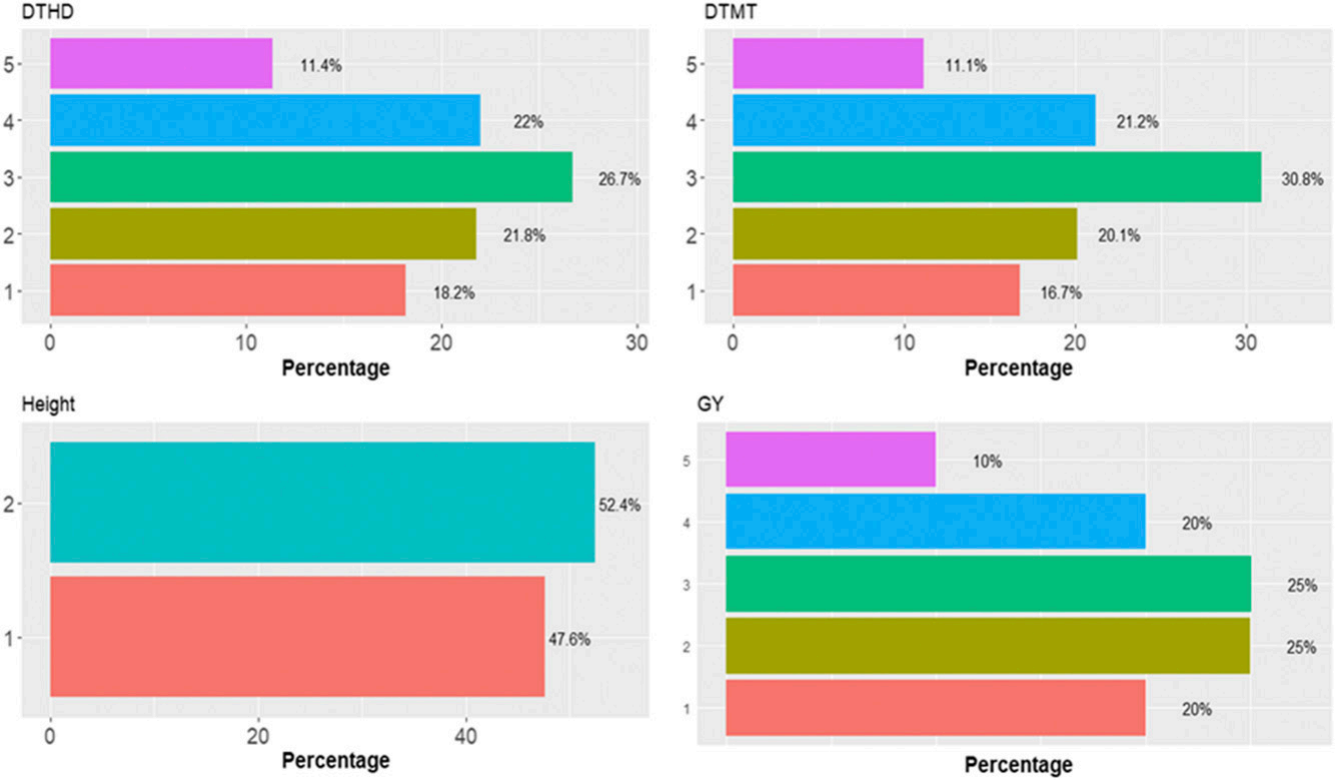
Percentage of individuals in each category of the ordinal response for *data set 4* across environments for traits days to heading (DTHD), days to maturity (DTMT) and Height.

**Figure 9:**
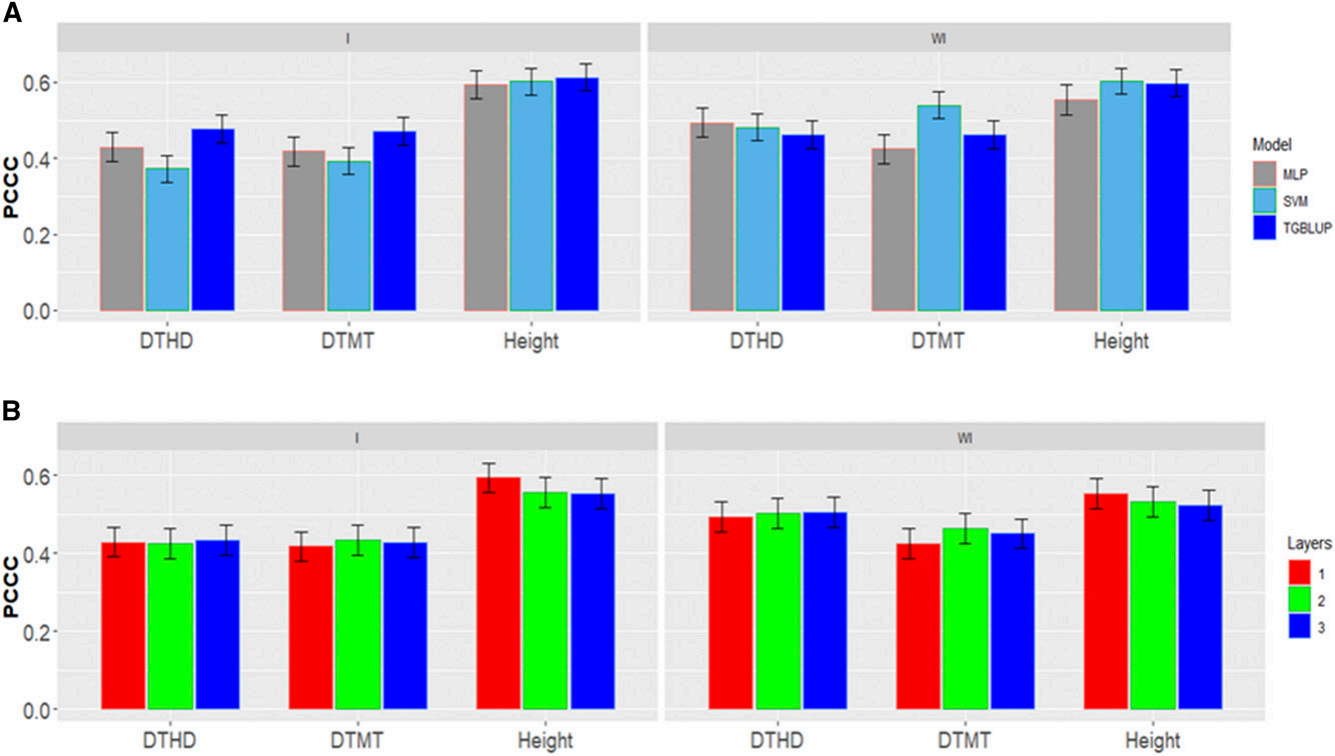
*Data set 4* in terms of percentage of cases correctly classified (PCCC) for traits days to heading (DTHD), days to maturity (DTMT) and Height. (A) Prediction accuracy of TGBLUP, MLP with one layer and SVM models with the G×E term (I) and without the G×E term (WI) for each trait; (B) prediction accuracy with different numbers of layers (1, 2 and 3) across environments with the MLP model with the G×E term (I) and without the G×E term (WI).

In Figure 8 it is observed that there are different percentages of individuals in each category in data set 4. For this data set, Figure 9 gives the prediction accuracies in terms of PCCC for each of the three traits evaluated under the three models (TGBLUP, SVM and MLP) with (I) and without (WI) the genotype × environment interaction term. When the genotype × environment interaction term was taken into account, the predictions ranged between 0.3720 and 0.6131, and the TGBLUP model produced the best predictions. However, when the genotype × environment interaction term was ignored, the predictions ranged between 0.4247 and 0.6028, and the best predictions were observed under the SVM method. We did not find statistical differences between using 1, 2 and 3 layers under the MLP model, which means that for this data set even the simple MLP model is enough for producing competitive predictions (Figure 9).

### Data set 5

**Figure 10:**
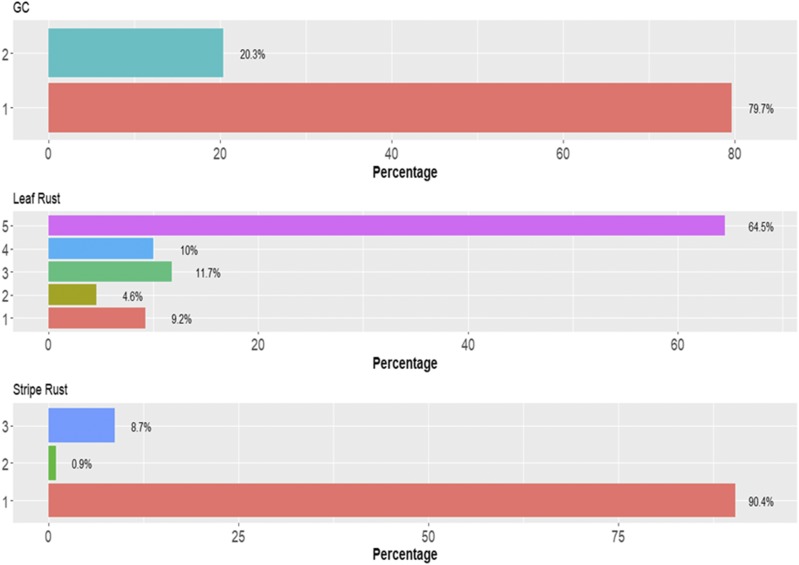
Percentage of individuals in each category of the ordinal response for *data set 5* for traits grain color (GC), Leaf Rust and Stripe Rust.

**Figure 11:**
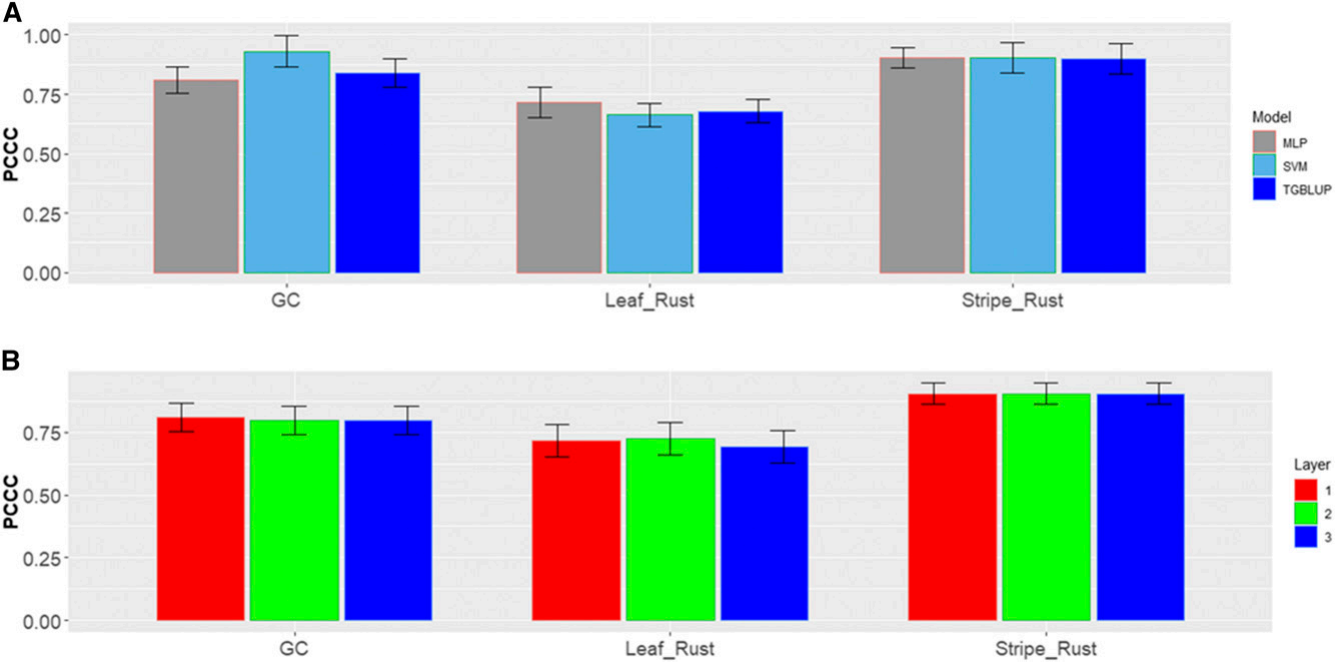
*Data set 5* in terms of percentage of cases correctly classified (PCCC) for traits grain color (GC), leaf rust and stripe rust. (A) Prediction accuracy of TGBLUP, SVM and MLP with one layer; (B) prediction accuracy with different numbers of layers (1, 2 and 3) across environments with the MLP model.

There are different percentages of individuals in each category in data set 5 (Figure 10). Figure 11 gives the prediction accuracies in terms of PCCC for the three traits under study under the three models (TGBLUP, SVM and MLP). We can see that for trait grain color (GC), the best prediction occurred under the SVM method and the worst under the MLP model; however, there were no significant differences between the three methods in any of the three traits under study, and the predictions ranged between 0.5082 and 0.5273. Also, it is important to point out that no significant differences were found for the MLP model with 1, 2 and 3 hidden layers (Figure 11).

### Data set 6

**Figure 12:**
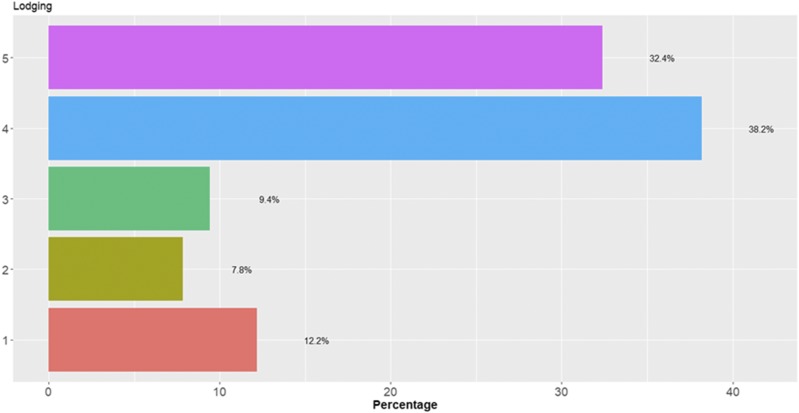
Percentage of individuals in each category of the ordinal response (Lodging) for *data* set 6.

**Figure 13:**
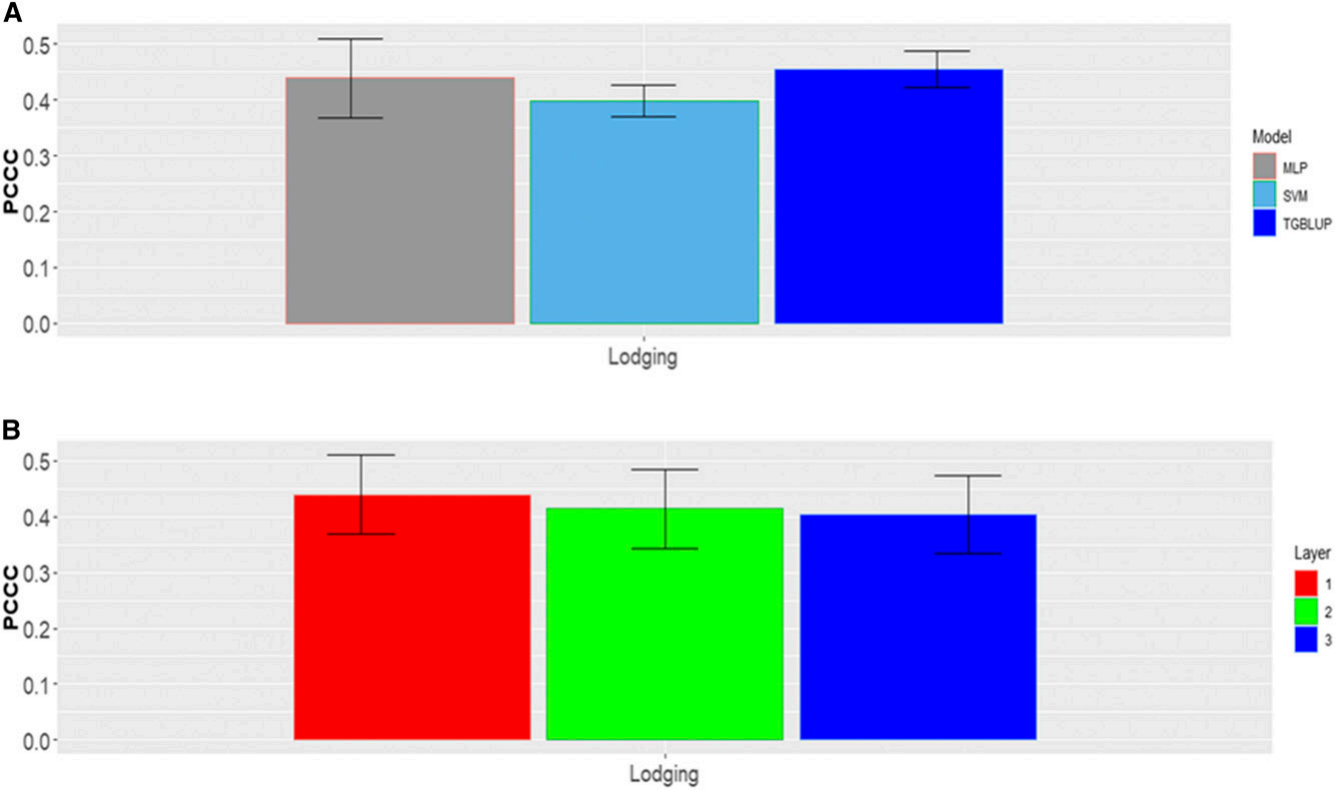
*Data set 6* in terms of percentage of cases correctly classified (PCCC) for trait Lodging. (A) Prediction accuracy of TGBLUP, SVM and MLP with one layer; (B) prediction accuracy with different numbers of layers (1, 2 and 3) across environments with the MLP model.

In each category there are different percentages of individuals for data set 6 (Figure 12). For this data set, Figure 13 does not show statistical differences between the three methods under study, but the predictions of TGBLUP were better than the predictions of the SVM and MLP models by 12.54% and 3.47%, respectively, while the MLP model was superior to the SVM model by 9.07%. The predictions ranged between 0.3979 and 0.4550. Figure 13 also shows that no significant differences were found using 1, 2 and 3 layers with the MLP model (Figure 13).

### Data set 7

**Figure 14:**
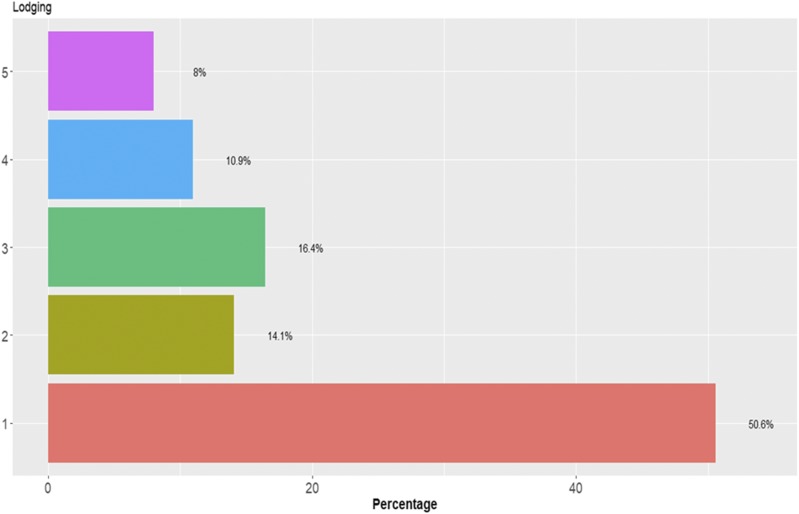
Percentage of individuals in each category of the ordinal response (Lodging) for *data* set 7.

**Figure 15:**
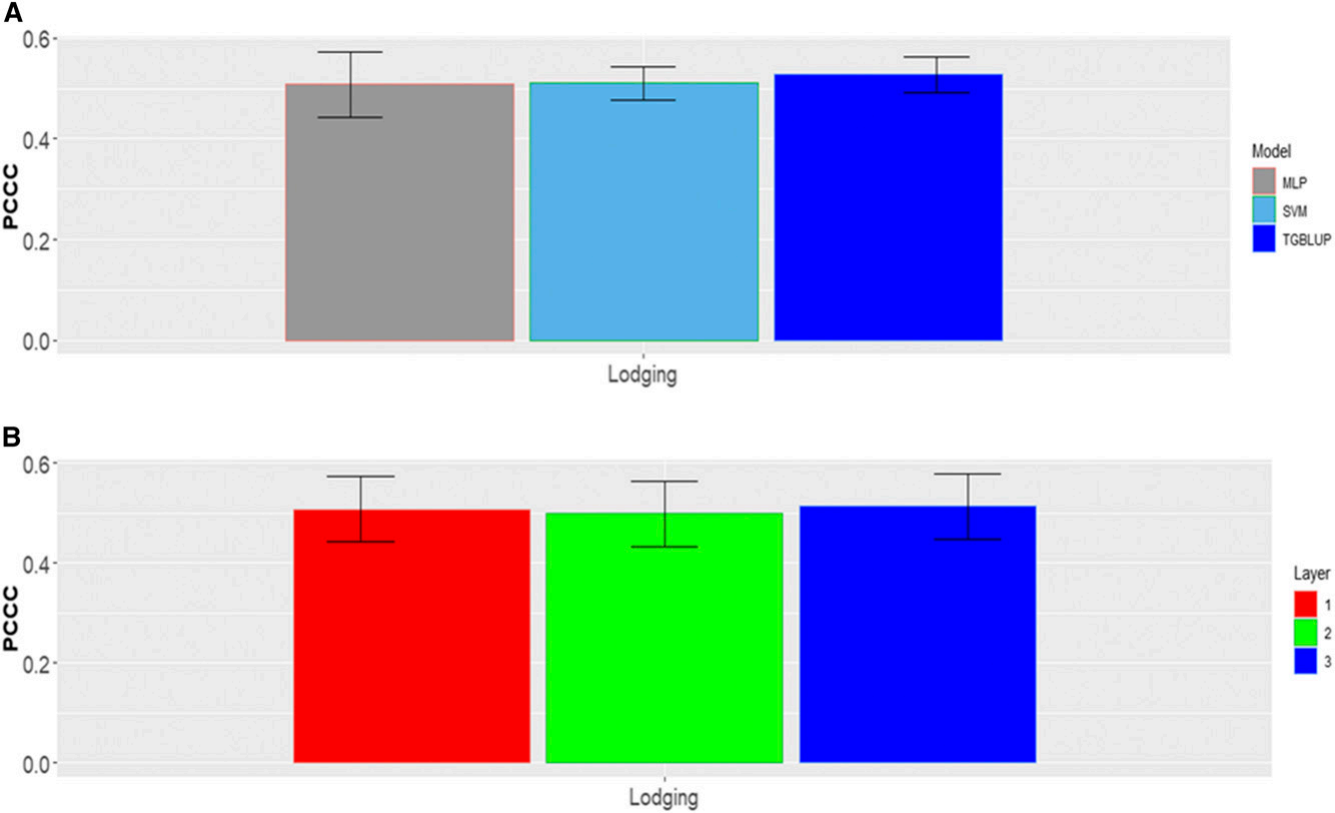
*Data set 7* in terms of percentage of cases correctly classified (PCCC) for trait Lodging. (A) Prediction accuracy of TGBLUP, SVM and MLP with one layer; (B) prediction accuracy with different numbers of layers (1, 2 and 3) across environments with the MLP model.

In each category there are different percentages of individuals for data set 7 (Figure 14). For this data set, no statistical differences were found between the three models (TGBLUP, SVM and MLP) for the trait Lodging, but the TGBLUP model was slightly better than the SVM and MLP models by 3.28% and 3.62%, respectively (Figure 15). The predictions for this trait under the three methods ranged between 0.5082 and 0.5273, respectively. Finally, also in this data set we did not find significant differences between using 1, 2 and 3 layers in the MLP model (Figure 15).

### Meta-comparison across environments and traits

**Figure 16:**
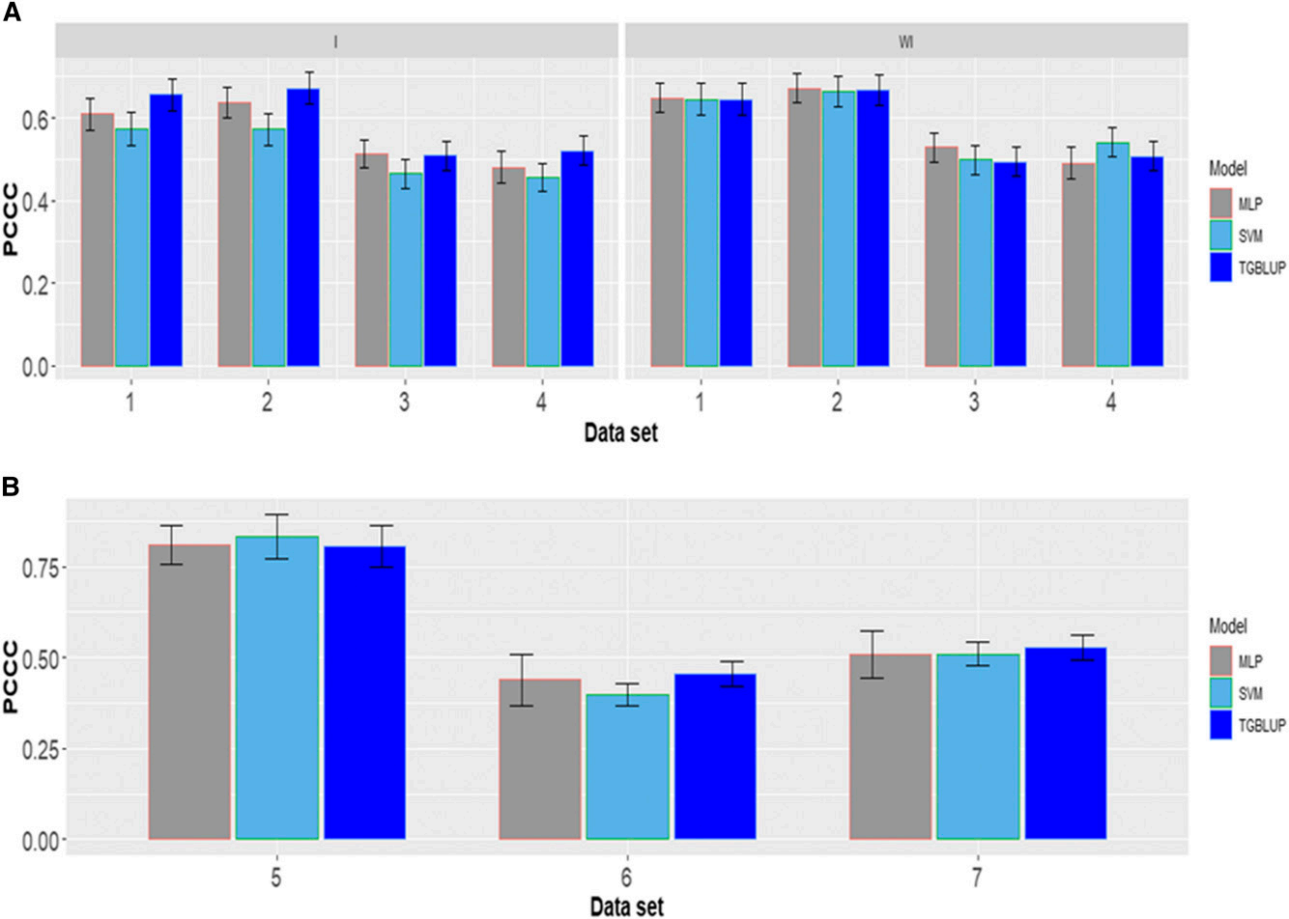
A meta-comparison across traits of the seven data sets in terms of percentage of cases correctly classified (PCCC) for the three methods under study (TGBLUP, SVM and MLP).

Finally, Figure 16 provided a meta-picture across environments and traits. When the interaction term was taken into account in the four data sets (1, 2, 3, and 4) the TGBLUP presented the best prediction accuracies, but not significantly differences were observed with those of the MLP model. While when the interaction term was ignored the three methods in the first four data sets perform very similar in terms of prediction performance. Also, in the meta-picture of data sets 5, 6 and 7 we found only in data set 6 a better performance of TGBLUP model but not statistical differences were found with the other two methods (SVM and MLP).

## Discussion

Due to the need for more powerful prediction models in the GS context to improve the selection process of candidate individuals in plant (or animal) breeding programs, we performed a benchmark study between two popular ML models (MLP and SVM) and the TGBLUP model with ordinal response variables (ordinal traits). This benchmark study is very important because many claim that ML methods outperform conventional genomic selection models in terms of prediction accuracy; however, there is not enough empirical evidence in the literature to support this claim. As stated by Makridakis *et al.* (2018), simply because the models are new or based on ML (or artificial intelligence) is not enough to persuade users of their practical advantages over alternative or conventional methods. For this reason, it is of paramount importance to properly evaluate the performance of ML methods using a wide range of diverse data sets and compare them to alternative or conventional models in order to obtain enough evidence of their prediction performance. For this reason, in this publication we used seven real data sets for evaluating the prediction performance of two machine learning methods, the MLP and SVM methods, against the TGBLUP model. We found that in general (4 out of 7 data sets), the best performance in terms of prediction accuracy for ordinal data using as a metric the percentage of cases correctly classified was obtained with the TGBLUP model, a conventional Bayesian method with weakly informative priors. However, it is important to point out that the predictions of both machine ML methods (MLP and SVM) were very competitive with the predictions of the TGBLUP model but not better, since the MLP method outperformed the TGBLUP model in two of the seven data sets under study, while the SVM method only outperformed it in one of the seven data sets used (across environments). For this reason, we agree with Makridakis *et al.* (2018), who pointed out that it should become clear that ML methods are not a panacea that would automatically improve forecasting accuracy. Their capabilities can easily generate implausible solutions, leading to exaggerated claims of their potential and must be carefully investigated before any claims can be accepted. This latter claim that ML methods can easily generate implausible solutions is supported by the fact that their successful application depends strongly on the tuning process, which is very challenging, so that when this process is not appropriate, the resulting solutions are very implausible.

Also, it is important to point out that for convenience we discretized the response variables in four out of the 7 data sets. However, there is evidence that the process of discretization of continuous variables produces a significant loss of information and this was studied by Kizikaya *et al.* (2014). When comparing the prediction accuracy using a threshold model for ordinal data and a model for continuous response variable using a data set with an ordinal response variable, Kizikaya *et al.* (2014) showed that to reach the same level of predictive capacity, the model of continuous response variable needs a training sample 2.25 times higher than when the threshold model is implemented. Also, Kizikaya *et al.* (2014) showed that using the same size of the training sample (TRN) the model for continuous response variable produces lower predictive capacity (16% lower) than using a threshold model.

A favorable attribute of many supervised learning algorithms is that there is no restriction on the distribution of response variables; this makes them less sensitive to the problems that arise in parametric models when ordinal scores are used to quantify diseases. Our results found using the MLP, the SVM methods, and the Bayesian threshold genomic best linear unbiased prediction (TGBLUP) model using ordinal data are similar to those found by other authors. For example, Ornella *et al.* (2012) compared the performance of ridge regression with Bayesian Lasso and two Support Vector Regression for predicting ordinal traits stem rust and yellow rust resistance in five wheat populations. The Bayesian Lasso and the Ridge Regression had similar prediction performance and with a small superiority over Support Vector Regression. González-Camacho *et al.* (2018) compared the performance of several regression/classification ML models against some parametric models (Bayesian LASSO, ridge regression, etc.) on stem rust and yellow rust and found similar results as those found by Ornella *et al.* (2012).

Our results support the idea that the ML methods are not the panacea for prediction modeling and we invite the users of prediction models not to blindly adopt or accept any model and assume it is the best in terms of prediction performance, since more empirical evidence is needed and a lot of research supports the idea that there is no universal model or machine (Montesinos-López *et al.*, 2018a,b,c; Bellot *et al.*, 2018). However, we need to be willing to test and adopt models coming from other areas since we need to improve the prediction accuracy of existing GS models to be able to really revolutionize plant and animal breeding and produce the required amount of food that the world needs and will need in the coming years without bringing more land under cultivation.

Also, we are convinced that more benchmark studies with a wide range of real data sets should be conducted to be able to clearly identify those models that perform better in terms of prediction accuracy (Benjamin, *et al.*, 2018). When researchers propose new models for prediction, they should compare their models to conventional models and use many data sets so as to provide enough evidence that the new proposed models actually outperform conventional models, since a lot of the new proposed models are not better than conventional models in terms of prediction accuracy. However, many times the new models are more complex to understand and to implement, which does not really help satisfy the need for more powerful prediction models and for advancing in the field of prediction modeling.

Another disadvantage of the ML methods is that a preprocessing step is needed to be able to apply them successfully, since the results depend on the type of transformation or preprocessing that is used (Chollet and Allaire 2017). For this reason, ML methods need to be improved to perform the tuning process and choose the appropriate preprocessing step automatically to be able to implement these methods more easily and with less possibility of error due to the fact that the tuning process and preprocessing step are very challenging.

Finally, with the seven real data sets used we found that the ML models (SVM and MLP) are very competitive with the TGBLUP model and can be implemented in the GS context since software in the ML domain has been developed that is very easy to use and works reasonably well with moderately large data sets. For this reason, we encourage other scientists to perform more benchmark studies and compare the existing GS models to ML methods, since the ML community had developed many efficient and friendly software to implement prediction models that can be used successfully in GS. We are convinced that by exploring the theory behind ML methods, we will find a lot of opportunities to improve the prediction performance of these methods and those of GS since this area had long time working in developing prediction models.

### Conclusions

In this paper, we explored two very popular machine learning methods, the MLP and SVM models, and compared them to the TGBLUP model in terms of prediction performance of ordinal data using seven real data sets. We found that in general (four out of seven data sets), the TGBLUP model was the best in terms of prediction accuracy using the percentage of cases correctly classified as a metric, followed by the MLP and SVM models. However, the two machine learning methods were very competitive since they produced very similar predictions to those of the TGBLUP model and, in some cases, outperformed the TGBLUP model. The disadvantage of both machine learning methods is that, to produce reasonable predictions, they require a tuning process that is challenging since it is both an art and a scientific process. However, despite these difficulties, we found that both machine learning algorithms are very competitive and practical to implement in GS because they are easy to implement using the existing software and work efficiently with moderately large data sets.

## Appendix

**Table A1:** Average percentage of cases correctly classified (PCCC) for each data set, model, layer, type of interaction terms with genotype×environment (I) and without genotype×environment (WI), and trait DTHD = days to heading; DTMT = days to maturity; Height = plant height). SE denotes standard error. For data sets 1-4, the averages are across environments

Data set	Model	Layers	Interaction	Trait	PCCC	SE
1	SVM	_	I	DTHD	0.5611	0.0401
1	SVM	_	I	DTMT	0.5206	0.0403
1	SVM	_	I	Height	0.6408	0.0387
1	SVM	_	WI	DTHD	0.6644	0.0381
1	SVM	_	WI	DTMT	0.6049	0.0394
1	SVM	_	WI	Height	0.6621	0.0382
1	TGBLUP	_	I	DTHD	0.6773	0.0376
1	TGBLUP	_	I	DTMT	0.6206	0.0392
1	TGBLUP	_	I	Height	0.672	0.0378
1	TGBLUP	_	WI	DTHD	0.664	0.038
1	TGBLUP	_	WI	DTMT	0.5992	0.0395
1	TGBLUP	_	WI	Height	0.6722	0.0379
1	MLP	1	I	DTHD	0.6048	0.0378
1	MLP	1	I	DTMT	0.5697	0.0387
1	MLP	1	I	Height	0.6511	0.0359
1	MLP	1	WI	DTHD	0.6629	0.0353
1	MLP	1	WI	DTMT	0.6184	0.0373
1	MLP	1	WI	Height	0.6648	0.0352
1	MLP	2	I	DTHD	0.6006	0.038
1	MLP	2	I	DTMT	0.572	0.0387
1	MLP	2	I	Height	0.6571	0.0356
1	MLP	2	WI	DTHD	0.6708	0.0349
1	MLP	2	WI	DTMT	0.6218	0.0372
1	MLP	2	WI	Height	0.6436	0.0362
1	MLP	3	I	DTHD	0.606	0.0378
1	MLP	3	I	DTMT	0.5703	0.0388
1	MLP	3	I	Height	0.6472	0.036
1	MLP	3	WI	DTHD	0.668	0.0351
1	MLP	3	WI	DTMT	0.6167	0.0374
1	MLP	3	WI	Height	0.6388	0.0364
2	SVM	_	I	DTHD	0.5439	0.04
2	SVM	_	I	DTMT	0.5297	0.0401
2	SVM	_	I	Height	0.6428	0.0385
2	SVM	_	WI	DTHD	0.6834	0.0372
2	SVM	_	WI	DTMT	0.6386	0.0384
2	SVM	_	WI	Height	0.6698	0.0377
2	TGBLUP	_	I	DTHD	0.7021	0.0365
2	TGBLUP	_	I	DTMT	0.639	0.0384
2	TGBLUP	_	I	Height	0.6751	0.0376
2	TGBLUP	_	WI	DTHD	0.6937	0.0368
2	TGBLUP	_	WI	DTMT	0.6371	0.0384
2	TGBLUP	_	WI	Height	0.6728	0.0376
2	MLP	1	I	DTHD	0.6266	0.0367
2	MLP	1	I	DTMT	0.6071	0.0374
2	MLP	1	I	Height	0.6764	0.0344
2	MLP	1	WI	DTHD	0.697	0.0331
2	MLP	1	WI	DTMT	0.6481	0.0357
2	MLP	1	WI	Height	0.6696	0.0347
2	MLP	2	I	DTHD	0.6211	0.037
2	MLP	2	I	DTMT	0.6005	0.0377
2	MLP	2	I	Height	0.6725	0.0346
2	MLP	2	WI	DTHD	0.6972	0.033
2	MLP	2	WI	DTMT	0.6403	0.036
2	MLP	2	WI	Height	0.6698	0.0347
2	MLP	3	I	DTHD	0.6175	0.0371
2	MLP	3	I	DTMT	0.6075	0.0375
2	MLP	3	I	Height	0.6653	0.035
2	MLP	3	WI	DTHD	0.6882	0.0336
2	MLP	3	WI	DTMT	0.6368	0.0363
2	MLP	3	WI	Height	0.6636	0.0351
3	SVM	_	I	DTHD	0.3771	0.0346
3	SVM	_	I	DTMT	0.363	0.0344
3	SVM	_	I	Height	0.6535	0.0342
3	SVM	_	WI	DTHD	0.4508	0.0357
3	SVM	_	WI	DTMT	0.4059	0.0352
3	SVM	_	WI	Height	0.6377	0.0346
3	TGBLUP	_	I	DTHD	0.4699	0.0358
3	TGBLUP	_	I	DTMT	0.4082	0.0354
3	TGBLUP	_	I	Height	0.6486	0.0344
3	TGBLUP	_	WI	DTHD	0.4526	0.0358
3	TGBLUP	_	WI	DTMT	0.3998	0.0352
3	TGBLUP	_	WI	Height	0.6307	0.0347
3	MLP	1	I	DTHD	0.4495	0.0343
3	MLP	1	I	DTMT	0.4259	0.0341
3	MLP	1	I	Height	0.6636	0.0314
3	MLP	1	WI	DTHD	0.5062	0.0349
3	MLP	1	WI	DTMT	0.441	0.0346
3	MLP	1	WI	Height	0.6377	0.0326
3	MLP	2	I	DTHD	0.4531	0.0345
3	MLP	2	I	DTMT	0.4221	0.0342
3	MLP	2	I	Height	0.6633	0.0314
3	MLP	2	WI	DTHD	0.4971	0.0349
3	MLP	2	WI	DTMT	0.4444	0.0347
3	MLP	2	WI	Height	0.631	0.0328
3	MLP	3	I	DTHD	0.4306	0.0339
3	MLP	3	I	DTMT	0.4214	0.034
3	MLP	3	I	Height	0.6632	0.0314
3	MLP	3	WI	DTHD	0.4653	0.0344
3	MLP	3	WI	DTMT	0.412	0.0339
3	MLP	3	WI	Height	0.6292	0.0329
4	SVM	_	I	DTHD	0.372	0.0345
4	SVM	_	I	DTMT	0.3922	0.0348
4	SVM	_	I	Height	0.6021	0.0348
4	SVM	_	WI	DTHD	0.4818	0.0357
4	SVM	_	WI	DTMT	0.5397	0.0347
4	SVM	_	WI	Height	0.6028	0.0349
4	TGBLUP	_	I	DTHD	0.4776	0.0356
4	TGBLUP	_	I	DTMT	0.4712	0.0354
4	TGBLUP	_	I	Height	0.6131	0.0347
4	TGBLUP	_	WI	DTHD	0.4621	0.0356
4	TGBLUP	_	WI	DTMT	0.4622	0.0353
4	TGBLUP	_	WI	Height	0.5984	0.035
4	MLP	1	I	DTHD	0.4291	0.0383
4	MLP	1	I	DTMT	0.418	0.0378
4	MLP	1	I	Height	0.5934	0.0376
4	MLP	1	WI	DTHD	0.4936	0.0391
4	MLP	1	WI	DTMT	0.4247	0.0378
4	MLP	1	WI	Height	0.5541	0.0385
4	MLP	2	I	DTHD	0.4242	0.0382
4	MLP	2	I	DTMT	0.4336	0.0382
4	MLP	2	I	Height	0.5553	0.0385
4	MLP	2	WI	DTHD	0.502	0.0391
4	MLP	2	WI	DTMT	0.4638	0.0381
4	MLP	2	WI	Height	0.5327	0.0389
4	MLP	3	I	DTHD	0.4331	0.0384
4	MLP	3	I	DTMT	0.4282	0.0381
4	MLP	3	I	Height	0.5531	0.0386
4	MLP	3	WI	DTHD	0.5062	0.039
4	MLP	3	WI	DTMT	0.4509	0.038
4	MLP	3	WI	Height	0.5237	0.039
5	SVM	_	_	GC	0.9315	0.0671
5	SVM	_	_	Leaf_Rust	0.6629	0.0477
5	SVM	_	_	Stripe_Rust	0.9035	0.0651
5	TGBLUP	_	_	GC	0.8392	0.0604
5	TGBLUP	_	_	Leaf_Rust	0.6785	0.0489
5	TGBLUP	_	_	Stripe_Rust	0.8994	0.0648
5	MLP	1	_	GC	0.8091	0.0555
5	MLP	1	_	Leaf_Rust	0.7158	0.0637
5	MLP	1	_	Stripe_Rust	0.9035	0.0417
5	MLP	2	_	GC	0.7977	0.0567
5	MLP	2	_	Leaf_Rust	0.7241	0.0631
5	MLP	2	_	Stripe_Rust	0.9035	0.0417
5	MLP	3	_	GC	0.7967	0.0568
5	MLP	3	_	Leaf_Rust	0.6919	0.0652
5	MLP	3	_	Stripe_Rust	0.9035	0.0417
6	SVM	_	_	Lodging	0.3979	0.0289
6	TGBLUP	_	_	Lodging	0.455	0.0331
6	MLP	1	_	Lodging	0.4392	0.0708
6	MLP	2	_	Lodging	0.4138	0.0702
6	MLP	3	_	Lodging	0.4042	0.07
7	SVM	_	_	Lodging	0.51	0.0336
7	TGBLUP	_	_	Lodging	0.5273	0.0348
7	MLP	1	_	Lodging	0.5082	0.0646
7	MLP	2	_	Lodging	0.4987	0.0646
7	MLP	3	_	Lodging	0.5134	0.0646
